# Implicit Sexual Attitude of Heterosexual, Gay and Bisexual Individuals: Disentangling the Contribution of Specific Associations to the Overall Measure

**DOI:** 10.1371/journal.pone.0078990

**Published:** 2013-11-18

**Authors:** Pasquale Anselmi, Michelangelo Vianello, Alberto Voci, Egidio Robusto

**Affiliations:** Department FISPPA, University of Padua, Padua, Italy; The University of New South Wales, Australia

## Abstract

The article aims to measure implicit sexual attitude in heterosexual, gay and bisexual individuals. A Many-Facet Rasch Measurement analysis was used to disentangle the contribution of specific associations to the overall IAT measure. A preference for heterosexuals relative to homosexuals is observed in heterosexual respondents, driven most by associating positive attributes with heterosexuals rather than negative attributes with homosexuals. Differently, neither the negative nor the positive evaluation of any of the target groups play a prominent role in driving the preference for homosexuals observed in gay respondents. A preference for heterosexuals relative to homosexuals is observed in bisexual respondents, that results most from ascribing negative attributes to homosexuals rather than positive attributes to heterosexuals. The results are consistent with the expression of the need for achieving a positive self-image and with the influence of shared social norms concerning sexuality.

## Introduction

Sexual prejudice refers to a negative attitude toward an individual because of her or his sexual orientation. Given the widely shared perception of heterosexuality as the “correct” way of intending sexual behavior, this type of prejudice is often specifically directed toward people who engage in homosexual behaviors, and thus toward gay men, lesbians, or bisexual individuals [1].

In the last decade, research on prejudice based on sexual orientation has taken advantage of the diffusion of implicit measures. These measures aim at capturing positive and negative features of the target object that might rely outside of conscious awareness, or that might not be accurately reported through introspection. For these reasons, implicit measures are more resistant than explicit measures to response biases, such as social desirability, impression management strategies and limited introspective access [2].

The Sexuality Implicit Association Test (Sexuality IAT) [3] has been the most widely used implicit measure of sexual attitude. It is a computerized two-choice discrimination task in which stimuli have to be categorized as belonging to the target categories *Heterosexuals* and *Homosexuals*, or to the attribute categories *Good* and *Bad* by pressing, as quickly and accurately as possible, one of two response keys. Categorizing the stimuli more quickly when *Heterosexuals* shares the response key with *Good* – and *Homosexuals* with *Bad* – than when it shares the response key with *Bad* – and *Homosexuals* with *Good* – is taken to indicate an implicit preference for heterosexuals relative to homosexuals.

Previous research using the Sexuality IAT found that individuals tend to prefer heterosexuals to homosexuals [3]–[6]. Looking at participants with different sexual orientation, a preference for one's own sexual orientation was observed in both heterosexual and gay individuals [7], [8], although such a preference was weaker for the latter [9] (see [10] for a review).

To date, however, there are no studies specifically focused on the investigation of the implicit sexual attitude of bisexual individuals. When present [4], [5], data concerning bisexuals were aggregated with those regarding gays. In this paper, we investigated the implicit sexual attitude of heterosexual, gay and bisexual individuals, keeping the three groups distinct.

As an additional novelty, we analyzed the contribution of specific associations between target groups and positive vs. negative attributes, using a methodology that allowed us to decompose the results of a Sexuality IAT. The IAT effect is the comparison between the response latencies in two mappings that differ in the target and attribute categories that share the same response key. There is a growing interest on analyzing and decomposing the IAT effect. For instance, models such as the QUAD model [11], the diffusion model [12], and the discrimination-association model [13] have been proposed that disentangle multiple, qualitatively different processes underlying the responses to the IAT. In the present paper, we disentangled the contribution of specific associations to the overall measure by investigating how the speed of categorization of individual stimuli changed according to the associative condition they were presented in. It is worth noting that the proposed procedure is different from sorting the trials into subsets and computing separate IAT effects for the two targets. Such a procedure is not advisable in analyzing IAT data [14]. Instead, we assessed the differential contribution of individual stimuli to the overall IAT effect, which remains a measure of a target relative to the other. The analysis was conducted through a Many-Facet Rasch Measurement (MFRM) [15]. Being a Rasch model [16], the MFRM provides us with a rigorous measurement system [17]. The speed of categorization of the stimuli is expressed by interval measures characterized by a measurement unit that, if the data fit the model, maintains the same size over the entire continuum. It follows that the measurement and comparison of different elements is more precise. Moreover, the MFRM allows us to investigate whether the speed of categorization of individual stimuli differs in the two associative conditions.

The proposed procedure has a strong potential for understanding the meaning of the implicit measure itself. For instance, consider the implicit preference for white people relative to black people that is usually observed in the literature [3], [18]. If the stimuli that contribute most to the measure are the positive ones, rather than the negative ones, then the implicit preference should be interpreted more as an expression of pro-Whites bias, rather than anti-Blacks bias. If the opposite occurs, the same implicit preference should be interpreted more as an expression of anti-Blacks bias, rather than pro-Whites bias. Taking into account the contribution of different stimuli provides researchers with a detailed depiction of the implicit associations that mostly underlie the measure (and that might differ across individuals), and allows them to precisely define the construct under investigation. Moreover, it prevents researchers from misreading the real meaning of the implicit measure. An example of such a misunderstanding has been described in the analysis of racial attitude. By means of the proposed procedure, it was found that the implicit preference for white people displayed by white individuals especially resulted from the attribution of positive traits to Whites, rather than of negative traits to Blacks [19]. Therefore, it was argued that the implicit measure of racial attitude might not necessarily imply black derogation, but could be mostly related to white favoritism. Independent contributions reached similar conclusions with different strategies of analysis (see, e.g., [20], [21]).

Disentangling the contribution of individual stimuli could be particularly useful when sexual prejudice is measured across different sexual orientations. For instance, it might help to answer questions concerning the direction of the preference observed in heterosexuals (e.g., whether it is most related to a preference for heterosexuals or to a derogation of homosexuals), the possible different meaning of the preference for one's own sexual orientation usually showed by heterosexuals and gays, and the reasons underlying implicit preferences showed by bisexual individuals.

## Methods

### Ethics statement

The study was conducted online. Visitors to the Project Implicit Italian demonstration site (https://implicit.harvard.edu/implicit/italy) self-selected to participate in the “Sexuality IAT” task. Participants voluntarily searched for and accessed the Project Implicit demonstration site in order to assess associations they may have about people and social groups. Before entering the study, they were informed that the study might detect associations that they were not consciously aware of and with which they might even explicitly disagree. Before entering the study, participants gave their consent by clicking on the following sentence: “I am aware of the possibility of encountering interpretations of my IAT test performance with which I may not agree. Knowing this, I wish to proceed”. Participants were able to drop out of the study at any time without any consequences. The study required 10–15 minutes to complete and participants received feedback about their IAT performance at the end. The study was in Italian. In Italy there is no legal requirement to obtain approval from an institutional review board (IRB) for non-clinical research studies. The authors work in a university in which no IRB existed at the time when the data were collected.

### Respondents

Out of the 940 respondents who started the IAT in a two years long data collection, 895 provided complete data, and 789 provided data that were interpretable according to the data reduction criteria for internet research [22]. Their mean age was 27.73 (*SD* = 9.01; range from 13 to 70), and 391 (49.56%) were female. Six hundred eighty-nine respondents identified themselves as heterosexuals (mean age 27.51, *SD* = 9.00; 50.80% female), 47 as gays (mean age 30.40, *SD* = 9.77; 25.53% female), and 53 as bisexuals (mean age 26.71, *SD* = 8.05; 54.72% female).

### Materials and procedure

The Sexuality IAT used the category labels *Heterosexuals*, *Homosexuals*, *Good* and *Bad*. Images and words were used to represent the target categories *Heterosexuals* and *Homosexuals*. Positive words and negative words were used to represent the attribute categories *Good* (beautiful, glorious, joyful, lovely, marvelous, pleasure and wonderful) and *Bad* (agony, awful, horrible, humiliate, nasty, painful, terrible and tragic). We employed the Italian translation of these words, which are currently in use in the Sexuality IAT provided by Project Implicit (https://implicit.harvard.edu/implicit). Stimuli were presented in the center of the computer screen in an alternating fashion, and respondents had to categorize them by pressing, as quickly and accurately as possible, the response key *E* or *I*. A red cross appeared in the event of a mistake. The procedure consisted of seven blocks. Three practice blocks involved the categorization of stimuli that represented either the target categories or the attribute categories. Four critical blocks involved the simultaneous categorization of stimuli representing the four categories with two response mappings. In one mapping, *Heterosexuals* and *Good* shared a response key, and *Homosexuals* and *Bad* shared the other. In the other mapping, *Heterosexuals* and *Bad* shared a response key, and *Homosexuals* and *Good* shared the other. The order of the two mappings was counterbalanced across the respondents.

### Data analysis

The IAT data (available at http://www.openscienceframework.org/) were analyzed through a MFRM [15], a probabilistic model belonging to the family of Rasch models. The model represents a rigorous frame of reference [17] in which measuring and comparing the stimuli, and allows the investigation of the contribution of individual stimuli to the overall IAT measure.

The MFRM takes into account any source of systematic variability (facet) which might be useful for explaining the likelihood of a response. In the present study, facets are (a) respondents, (b) sexual orientation of respondents, (c) attribute stimuli, and (d) associative condition.

Responses smaller than 300 ms and greater than 10,000 ms were excluded from the analysis, and response times were discretized according to the tertiles computed on the 789 (number of respondents)×2 (number of associative conditions)×15 (number of attribute stimuli) complete data matrix (for a similar data analysis, see [19], [23], [24]). In our dependent variable, the values 3, 2 and 1 identify fast, medium and slow responses, respectively.

The MFRM analysis was performed using the computer program FACETS 3.70.0 [25]. A parameter α was estimated for each respondent indicating his/her speed in completing the IAT, a parameter β for heterosexual, gay and bisexual respondents indicating their speed, a parameter γ for each attribute stimulus indicating its speed of categorization, and a parameter ε for each associative condition indicating the ease of the condition. All the estimates are interval measures. Higher values indicate higher response speed, and they should be interpreted as higher respondents' speed in completing the IAT, higher speed of categorization of the stimuli, and greater ease of the associative conditions. Estimates of sexual orientations, associative conditions and attribute stimuli were constrained to have a mean element estimate of zero.

The MFRM analysis provided a number of indices (for details, see [26], [27]). Data-model infit and outfit statistics were computed for each element of each facet. Expected values for both statistics are equal to 1, and values in the range from 0.5 to 2 express good fit [25].

A fixed all same Chi-square (χ^2^) and a separation reliability (*R*) are computed for each facet. χ^2^ tests the null hypothesis that the differences between the measures of the elements of a facet are equal to zero. *R* ranges between 0 and 1, and shows how much the differences observed between the estimates are true and not due to error (*R* close to 1).

The MFRM allows the analysis of the interactions between elements of different facets [25]. The interaction between the facets associative condition and sexual orientation allowed us to investigate whether the ease of the associative conditions changes with the sexual orientation of respondents (differential condition functioning). Moreover, the interaction between the facets attribute stimuli, associative condition and sexual orientation allowed us to investigate whether the speed of categorization of the stimuli changes according to the sexual orientation of respondents and the associative condition they are presented in (differential stimulus functioning). This provides us with the contribution of each individual stimulus to the overall IAT measure and with a detailed depiction of the implicit associations underlying the responses provided by individuals of different sexual orientation.

## Results

Both infit and outfit were excellent for the facets sexual orientation (0.97≤infit/outfit≤1.28), attribute stimuli (0.83≤infit/outfit≤1.13), and associative condition (0.95≤infit/outfit≤1.06). Only 14 out of the 789 respondents (1.78%) had an infit or outfit outside the recommended range.

Respondents completed the IAT with different speeds (α ranges from −3.22 to 2.99; *R* = 0.87; χ^2^(788) = 4857.1, *p*<0.001). The stimuli were categorized with different speeds (γ ranges from −0.45 to 0.30; *R* = 0.97; χ^2^(14) = 423.9, *p*<0.001). The condition Heterosexuals-Good/Homosexuals-Bad (HE-G/HO-B) was easier than the condition Homosexuals-Good/Heterosexuals-Bad (HO-G/HE-B; ε_HE-G/HO-B_ = 0.47; ε_HO-G/HE-B_ = −0.47; *R* = 1; χ^2^(1) = 2494.9, *p*<0.001). The distance Δ = 0.94 between the two conditions represents the size of the IAT effect. It is significantly different from 0 (*z* = 66.47, *p*<0.001), meaning that, taken all together, respondents implicitly preferred heterosexuals to homosexuals.

Analyzing the IAT effect computed on respondents of different sexual orientation, we found that both heterosexual and bisexual respondents significantly preferred heterosexuals to homosexuals (Δ_heterosex_ = 1.08, *z*_heterosex_ = 76.37, *p*<0.001; Δ_bisex_ = 0.29, *z*_bisex_ = 4.10, *p*<0.001), but heterosexual respondents held a stronger preference than bisexual respondents (*z*_heterosex-bisex_ = 11.07, *p*<0.001). Gay respondents significantly preferred homosexuals to heterosexuals (Δ_gay_ = −0.20, *z*_gay_ = −2.83, *p*<0.01). To compare the strength of the preference observed in heterosexual and gay respondents, we compared the size of their IAT effects in absolute terms: heterosexual respondents held a stronger preference for their own sexual orientation than gay respondents (*z*_|heterosex|-|gay|_ = 12.21, *p*<0.001). For the sake of comparison with classical analysis, we also computed the traditional *D*_2_ score [28], obtaining the same pattern of results: mean *D*_heterosex_ = 0.52, *SD*_heterosex_ = 0.41; mean *D*_bisex_ = 0.16, *SD*_bisex_ = 0.48; mean *D*_gay_ = −0.16, *SD*_gay_ = 0.39.

Table 1 provides information concerning the differential stimulus functioning, separately for the three groups of respondents. For each individual stimulus, it is shown whether its overall speed of categorization (i.e., estimated across the two associative conditions) changes according to the specific associative condition the stimulus is presented in. This allowed us to investigate the contribution of each individual stimulus to the overall implicit measure.

**Table 1:** Speed of categorization of the stimuli in the two associative conditions for respondents of each sexual orientation.

	Heterosexuals-Good Homosexuals-Bad			Homosexuals-Good Heterosexuals-Bad						
Stimulus	ORS	MSR	SE	ORS	MSR	SE	*t*		*df*	Cohen's *d*
Heterosexuals (689)										
Pleasure	1590	0.10	0.06	1145	−0.16	0.06	3.06	**	1375	0.17
Marvelous	1567	0.02	0.06	1125	−0.23	0.06	2.95	**	1375	0.16
Wonderful	1527	−0.10	0.06	1103	−0.30	0.06	2.36	*	1374	0.13
Beautiful	1658	0.32	0.06	1261	0.20	0.05	1.54		1374	0.08
Terrible	1654	0.31	0.06	1269	0.22	0.05	1.15		1374	0.06
Awful	1577	0.07	0.06	1215	0.07	0.06	0.00		1372	0.00
Joyful	1601	0.13	0.06	1242	0.14	0.06	−0.12		1375	0.01
Humiliate	1510	−0.15	0.06	1155	−0.13	0.06	−0.24		1373	0.01
Horrible	1638	0.26	0.06	1291	0.29	0.05	−0.38		1374	0.02
Agony	1544	−0.05	0.06	1192	0.00	0.06	−0.59		1374	0.03
Glorious	1562	0.00	0.06	1235	0.12	0.06	−1.41		1375	0.08
Lovely	1389	−0.51	0.05	1080	−0.39	0.06	−1.54		1372	0.08
Painful	1584	0.08	0.06	1271	0.24	0.05	−2.05	*	1374	0.11
Nasty	1454	−0.32	0.05	1155	−0.12	0.06	−2.56	*	1374	0.14
Tragic	1502	−0.18	0.05	1218	0.07	0.06	−3.20	**	1375	0.17
Bisexuals (53)										
Humiliate	121	0.32	0.20	97	−0.29	0.20	2.16	*	103	0.43
Horrible	131	0.75	0.22	108	0.13	0.19	2.13	*	103	0.42
Tragic	123	0.40	0.20	106	0.05	0.19	1.27		103	0.25
Awful	114	0.05	0.19	102	−0.10	0.19	0.56		103	0.11
Lovely	103	−0.36	0.19	94	−0.41	0.20	0.18		103	0.04
Painful	117	0.17	0.20	109	0.16	0.19	0.04		103	0.01
Beautiful	120	0.28	0.20	112	0.27	0.19	0.04		103	0.01
Glorious	112	−0.03	0.19	104	−0.03	0.19	0.00		103	0.00
Terrible	112	−0.03	0.19	104	−0.03	0.19	0.00		103	0.00
Pleasure	110	−0.10	0.19	104	−0.02	0.19	−0.30		103	0.06
Agony	110	−0.10	0.19	108	0.13	0.19	−0.86		103	0.17
Marvelous	109	−0.14	0.19	108	0.12	0.19	−0.97		103	0.19
Wonderful	101	−0.43	0.19	101	−0.13	0.19	−1.12		104	0.22
Nasty	100	−0.47	0.19	102	−0.10	0.19	−1.38		103	0.27
Joyful	108	−0.17	0.19	109	0.23	0.20	−1.45		102	0.29
Gays (47)										
Glorious	97	0.31	0.21	87	−0.30	0.21	2.05	*	91	0.43
Painful	98	0.36	0.21	94	0.00	0.21	1.21		91	0.25
Lovely	81	−0.37	0.21	80	−0.61	0.21	0.81		91	0.17
Nasty	89	−0.02	0.21	88	−0.25	0.21	0.77		91	0.16
Terrible	101	0.49	0.21	103	0.38	0.21	0.37		91	0.08
Wonderful	81	−0.37	0.21	84	−0.42	0.21	0.17		91	0.04
Joyful	90	0.02	0.21	94	0.00	0.21	0.07		91	0.01
Awful	90	0.02	0.21	95	0.04	0.21	−0.07		91	0.01
Marvelous	94	0.19	0.21	102	0.34	0.21	−0.51		91	0.11
Humiliate	85	−0.11	0.21	95	0.04	0.21	−0.51		90	0.11
Horrible	100	0.45	0.21	109	0.66	0.22	−0.69		91	0.14
Tragic	81	−0.37	0.21	91	−0.13	0.21	−0.81		91	0.17
Pleasure	83	−0.28	0.21	94	0.00	0.21	−0.94		91	0.20
Agony	82	−0.32	0.21	94	0.00	0.21	−1.08		91	0.23
Beautiful	90	0.02	0.21	102	0.34	0.21	−1.08		91	0.23

*Note*. ORS = observed raw scores; MSR = measure. The *t* values test the hypothesis that the difference between the measures is equal to zero.

* *p*<0.05;

** *p*<0.01.

The speed with which heterosexual respondents categorized the positive stimuli *pleasure*, *marvelous* and *wonderful* increased in the condition Heterosexuals-Good/Homosexuals-Bad and decreased in the condition Homosexuals-Good/Heterosexuals-Bad, compared with the overall speed (i.e., across the two associative conditions) of these stimuli. Thus, heterosexual respondents associated these stimuli more easily with heterosexuals than with homosexuals. Differently, the speed with which heterosexual respondents categorized the negative stimuli *tragic*, *nasty* and *painful* increased in the condition Homosexuals-Good/Heterosexuals-Bad and decreased in the condition Heterosexuals-Good/Homosexuals-Bad. These were the stimuli that heterosexual respondents associated more similarly with heterosexuals and homosexuals. Therefore, *pleasure*, *marvelous* and *wonderful* were the stimuli that most contributed to increasing the implicit preference for heterosexuals observed in heterosexual respondents, whereas *tragic*, *nasty* and *painful* were the stimuli that most contributed to decreasing it. In gay respondents, only the stimulus *glorious* exhibited differential stimulus functioning. The speed of categorization of this stimulus increased in the condition Heterosexuals-Good/Homosexuals-Bad and decreased in the condition Homosexuals-Good/Heterosexuals-Bad. Gay respondents associated *glorious* more easily with heterosexuals than with homosexuals. With this single exception, the pattern of the different stimuli was similar and, in particular, there was no specific stimulus that mainly contributed to increasing the implicit preference for homosexuals shown by gay respondents.

Concerning bisexual respondents, the speed with which they categorized the negative stimuli *humiliate* and *horrible* increased in the condition Heterosexuals-Good/Homosexuals-Bad and decreased in the condition Homosexuals-Good/Heterosexuals-Bad: Bisexual respondents associated these stimuli more easily with homosexuals than with heterosexuals. Thus, *humiliate* and *horrible* were the stimuli that contributed most to increasing the implicit preference for heterosexuals relative to homosexuals observed in the bisexual respondents.

## Discussion

This article investigated the implicit sexual attitude of heterosexual, gay and bisexual individuals, analyzing the attributes that mostly drive implicit preferences. Consistently with the literature [7], [8], we observed that both heterosexual and gay respondents preferred individuals with their own sexual orientation, and that this preference was stronger for the former than for the latter. In addition, we found that bisexual respondents implicitly preferred heterosexuals to homosexuals. Looking at the contribution of individual stimuli, we found that the strong preference for their own sexual orientation observed in heterosexual participants was mostly driven by the attribution of positive traits to heterosexuals, rather than of negative traits to homosexuals. Differently, the weaker preference for homosexuals observed in gay respondents was not particularly guided by either positive or negative attributes. Finally, the preference for heterosexuals displayed by bisexual participants especially resulted from the attribution of negative traits to homosexuals, rather than of positive attributes to heterosexuals.

These results can be interpreted taking into account, at the same time, intergroup relations and shared social norms. The bias in sexual attitudes could be conceived as an intergroup phenomenon, in which people tend to evaluate their membership group more positively than other groups, with the aim of obtaining a positive social identity and, thus, a positive self-image [29]. This asymmetry is generally based on a particularly positive evaluation of the ingroup, while a negative evaluation of the outgroup is likely to occur only when this group is perceived as threatening [30], [31]. Concerning social norms, the widely shared beliefs regarding sexual orientations are claimed to lead to the perception of *heteronormativity*, defined as an internalized view according to which heterosexuality is the standard for legitimate and expected social and sexual relations [32], [33].

For heterosexuals, these two social processes point at the same direction: the self-image is protected and enhanced by a positive image of heterosexuality compared to homosexuality, and the shared social view based on heteronormativity is consistent with this perception. This could explain why heterosexual respondents showed a strong implicit preference for heterosexuality. Moreover, given that the perception of heterosexuality as natural and normal is considered stable, justified and not threatened [34], heterosexuals did not need to derogate homosexuals to obtain a positive image of their own sexual orientation.

For gay perceivers, however, the two processes may conflict. On the one hand, the homosexual ingroup could be preferred, as a way to increase self-esteem. On the other hand, shared beliefs related to heteronormativity could lead to biases in favor of heterosexuality: in this case, phenomena of self-stigma are possible, as individuals may internalize a negative view of their own group, and thus of themselves [35]. In our data, the need for a positive self-esteem could have led to ingroup preference, while self-stigma and heteronormativity perceptions could have weakened such a preference. The presence of opposite motives may explain why the global implicit preference was not particularly driven by individual attributes, either positive or negative.

Finally, for bisexual respondents, heteronormativity should not be counterbalanced by ingroup positivity, as in this case the very definition of the ingroup is unclear [36]. On the other hand, the presence of internalized self-stigma related to homosexual behaviors is likely [37]. This combination of motives might explain the implicit preference for heterosexuals, and the fact that it is driven by attributing the negative stimuli *humiliate* and *horrible* to homosexuals.

Although our findings are clear, some limitations about the present study should be noted. First, the physical absence of the experimenter in an online study increases the difficulty of ensuring that procedural instructions are clear to all respondents and that the task is performed in the proper way. However, it is reassuring that several online studies, employing different IATs, replicated the effects found in laboratory settings [3], [22]. Second, because of the method of data collection, respondents to the Sexuality IAT were self-selected and are not representative of any particular population. Second, the number of gay and bisexual respondents was not very large, in particular compared to the heterosexual sample. As a consequence, the obtained results should be replicated in further research.

Overall, our findings demonstrate the usefulness of considering, besides the usual global IAT scores, the specific contributions of positive and negative stimuli to the expression of implicit associations. The MFRM has proved to be a valid tool for this purpose. The model represents a rigorous frame of reference in which estimating and comparing the speed of categorization of the stimuli. Moreover, by allowing the analysis of differential stimulus functioning, the model provides us with the contribution of each stimulus to the overall IAT measure. Such an analysis is advisable when distinct and opposing drives are involved, as it can be the case of people with different sexual orientations. Disentangling the contribution of these drives could have important consequences for a clearer understanding of sexual prejudice and discrimination, with the aim of developing informed social policies for the reduction of these antisocial phenomena. Such a result is particularly desirable if we consider that gay and bisexual individuals, being targets of prejudice and discrimination, often face minority stress phenomena, with detrimental consequences on well-being, quality of life, and even mental health [38].
